# Physiology of pregnancy and oral local anesthesia considerations

**DOI:** 10.7717/peerj.15585

**Published:** 2023-06-29

**Authors:** Xueer Zhou, Yunyu Zhong, Zijian Pan, Jiankang Zhang, Jian Pan

**Affiliations:** 1 State Key Laboratory of Oral Disease, West China Hospital of Stomatology, Sichuan University, Chengdu, Sichuan, China; 2 National Clinical Research Center for Oral Diseases and Department of Oral and Maxillofacial Surgery, West China Hospital of Stomatology, Sichuan University, Chengdu, Sichuan, China; 3 Chengdu Advanced Medical Science Center, West China Hospital of Stomatology, Sichuan University, Chengdu, Sichuan, China

**Keywords:** Local anesthesia, Physiology of pregnancy, Oral treatments

## Abstract

**Background:**

Safe and effective local anesthesia is a prerequisite for emergency oral surgeries and most dental treatments. Pregnancy is characterized by complex physiological changes, and increased sensitivity to pain. Pregnant women are particularly vulnerable to oral diseases, such as caries, gingivitis, pyogenic granuloma and third molar pericoronitis. Maternally administered drugs can affect the fetus through the placenta. Therefore, many physicians and patients are reluctant to provide or accept necessary local anesthesia, which leads to delays in the condition and adverse consequences. This review is intended to comprehensively discuss the instructions for local anesthesia in the oral treatment of pregnant patients.

**Methodology:**

An in-depth search on Medline, Embase, and the Cochrane Library was performed to review articles concerned with maternal and fetal physiology, local anesthetic pharmacology, and their applications for oral treatment.

**Results:**

Standard oral local anesthesia is safe throughout the pregnancy. At present, 2% lidocaine with 1:200,000 epinephrine is considered to be the anesthetic agent that best balances safety and efficacy for pregnant women. Maternal and fetal considerations must be taken into account to accommodate the physiological and pharmacological changes in the gestation period. Semi-supine position, blood pressure monitoring, and reassurance are suggested for high-risk mothers to reduce the risk of transient changes in blood pressure, hypoxemia, and hypoglycemia. For patients with underlying diseases, such as eclampsia, hypertension, hypotension, and gestational diabetes, the physicians should use epinephrine cautiously and control the dose of anesthetic. New local anesthesia formulations and equipment, which contribute to minimizing injection pain and relieving the anxiety, have and are being developed but remain understudied.

**Conclusions:**

Understanding the physiological and pharmacological changes during pregnancy is essential to ensure the safety and efficiency of local anesthesia. Optimal outcomes for the mother and fetus hinge on a robust understanding of the physiologic alterations and the appropriate selection of anesthetic drugs and approaches.

## Introduction

Pregnancy is a particular period during which maintaining oral health is essential. Due to the existence of primary oral diseases and changes in the internal environment, the incidence of oral diseases increases during pregnancy (Cho et al., 2020; Choi et al., 2021; Mark, 2021). Although dental treatment during pregnancy has proven to be safe, due to lack of authoritative clinical consensus and guidelines (Lee & Shin, 2017; Manautou & Mayberry, 2023), many dentists are hesitant to provide dental therapy to pregnant women or even advise against any treatment during pregnancy (Lee et al., 2010). Furthermore, many pregnant women are afraid to seek dental treatment due to dental anxiety and neglect of the importance of oral health. Dental drills and anesthesia injections are the most likely factors for dental anxiety (Al Khamis et al., 2016; George et al., 2018; Liu et al., 2019; Rocha et al., 2018).

The mother and the fetus are connected *via* the placenta, and oral health care is particularly important for both. Current clinical practice guidelines clearly state that it’s not only safe, but also necessary to provide dental treatment during pregnancy (educational, preventive and restorative actions) (AAPD, 2023; ADA, 2021; CDA, 2010; Health MDoP, 2016; Mark, 2021; OHRC, 2023; WHO, 2017). Any improvement in women’s oral health and health education can impact children’s health (Figuero, Han & Furuichi, 2020; Fomete et al., 2021; Raju & Berens, 2021). Leaving severe inflammation and pain untreated during pregnancy can result in a more harmful emergency that may lead to general anesthesia surgery, hospitalization, and premature delivery (Bobetsis et al., 2020; Cho et al., 2020; Favero et al., 2021; Fomete et al., 2021; Iqbal et al., 2022; Kapila, 2021).

Local anesthesia is a prerequisite for most oral clinical treatments, which can relieve pain in dental procedures such as tooth extraction, fossa preparation, root canal treatment, abscess drainage, and minor oral surgery. Common crises in the oral clinic include syncope, hyperventilation, local anesthetic overdose, hypertension, hypoglycemia, and epileptic seizures, of which more than 50% are associated with local anesthesia injection (Smereka et al., 2019). Many local anesthetics and anesthesia methods are commonly used in the clinic, including 4% articaine and 3% mepivacaine infiltration anesthesia, 2% lidocaine, 0.5% bupivacaine, and 2% mepivacaine block anesthesia (Spivakovsky, 2019; St George et al., 2018; Wang et al., 2021). However, pregnancy causes many physiological changes, and maternally administered drugs can affect the fetus through the placenta. Therefore, the selection of anesthetics and anesthesia approaches should be deliberated for the perioperative and intraoperative management of pregnant patients. Herein, this article will comprehensively discuss the instructions for local anesthesia in the oral treatment of pregnant patients and clarify doubts of health professionals, in combination with the physiological changes during pregnancy, local anesthetics pharmacology, advanced materials and equipment, and the process of oral therapy.

## Survey methodology

An in-depth search on Medline, Embase, and the Cochrane Library was performed to review articles concerned with maternal and fetal physiology, local anesthetic pharmacology, and their applications for oral treatment. A broad range of keywords and phrases were searched, including “pregnancy”, “maternal”, “fetal”, “physiology”, “dental” and “oral”, “anesthesia”, “topical anesthesia”, “local anesthetic”, “epinephrine”, “lidocaine”, “efficacy”, “pediatric”, “pain”, “infection”, “buffer”, “warm”, and “equipment”, performed by crossing these descriptors using the Boolean operators “OR” and “AND”. In the process of summarizing the recommendations for the use of local anesthetics during pregnancy, we further reviewed relevant guidelines from various institutions. Gray literature (monographs, dissertations, theses, books, chapters, studies published in event proceedings) and studies without full text information were excluded. Additional references for possible inclusion were obtained through manual searching. A selection of titles was made, from which the abstracts were read and those that met the topic of the article were selected. A preliminary review of 546 studies was accomplished through electronic database searching, the majority of which were excluded after reviewing the title and abstract. The remaining 237 articles proceeded to the final full-text review and resulted in the inclusion of 182 studies.

### Physiology of pregnancy and anesthesia considerations

**Figure 1:**
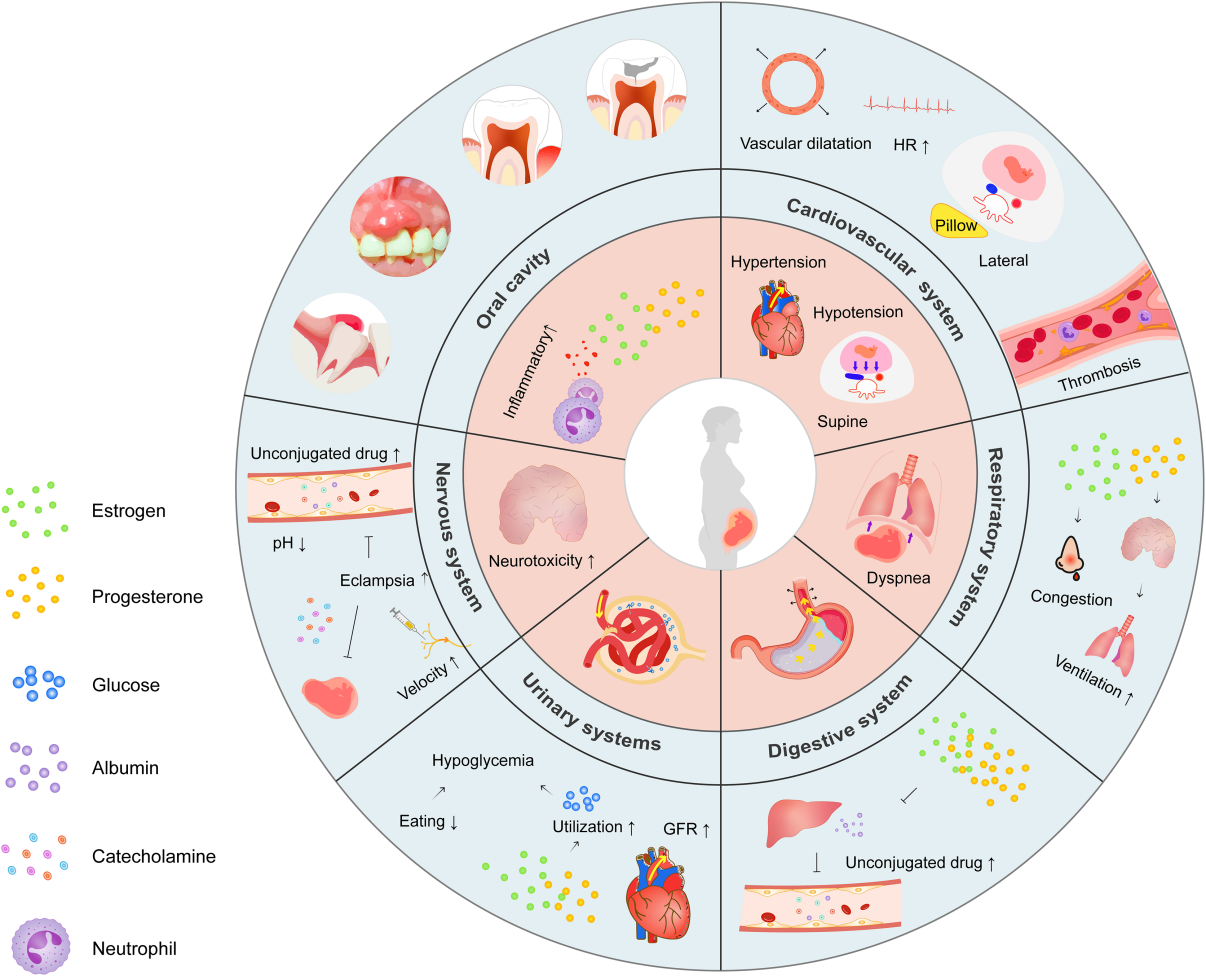
Schematic illustration of physiological changes during pregnancy. Overview highlighting the major physiological changes in each system (inner ring) and the mechanisms behind them (outer ring).

Pregnancy is characterized by physiological alterations necessary for fetal growth, some of which may affect local anesthesia efficiency (Fig. 1). Specifically, systemic changes with respect to the cardiovascular, hematologic, respiratory, and nervous systems require careful monitoring. Gastrointestinal, renal, and endocrine alterations may interfere with the determination of anesthesia dose.

### Cardiovascular and hematological alterations

The cardiac output in pregnant patients can increase by 50% in early pregnancy, attributed to peripheral vasodilation, decreased systemic vascular resistance, and a 20% to 30% increase in heart rate (Aleksenko & Quaye, 2020; Yanamandra & Chandraharan, 2012). Pregnancy is associated with increased sympathetic activity, which may contribute to arrhythmias (Tamirisa et al., 2022). As the heart is pushed upward and rotated forward, echocardiography often shows ventricular enlargement and myocardial hypertrophy (Aleksenko & Quaye, 2020). Pre-existing cardiovascular conditions such as pre-eclampsia or eclampsia may also progress dramatically during pregnancy.

During the second trimester, pregnant women are at higher risk of hypertension, clinically evident in 8% of pregnant patients (Barr et al., 2022), characterized by poorer oral health and a higher incidence of complications with local anesthesia (Mata et al., 2021). During dental treatment, fear and pain are the most common causes of high blood pressure. Although treatment of transient hypertension is not recommended in generally healthy patients, severe fluctuations in blood pressure should be avoided in pregnant patients, refraining from hypoxia caused by decreased cerebral and uterine perfusion (Papademetriou et al., 2021). If the pregnant woman’s intraoperative blood pressure suddenly increases, the physicians and dentists should stop the operation immediately, give oxygen in time and closely observe the patient’s systemic condition, eliminate the patient’s tension and anxiety, and consider whether to continue the treatment according to the patient’s recovery (Southerland et al., 2016).

Hypotension usually occurs during the second and third trimesters. Hypotension will lead to reduced blood flow to the uterus and fetal perfusion and even lead to fetal hypoxia, so it is necessary to monitor and maintain maternal blood pressure and fluid volume carefully. When the patient is in the supine position, the fetus significantly compresses the inferior vena cava and aorta. As a result, the venous return of cardiac blood volume is impaired, which results in a 14% reduction in stroke output. Blood pressure and cardiac output are also decreased, resulting in oxygen deprivation to the brain and uterus, which may lead to supine hypotension syndrome. Supine hypotension syndrome occurs in approximately 10% of pregnant patients and is usually characterized by hypotension, syncope, and bradycardia (Massoth et al., 2022). The patient should be placed in a semi-supine position or told to lean 5° to 15° to the left (right hip elevation 10 to 12 cm) with a pad on the right lower back to move the uterus toward the aorta, which is less prone to collapse. In addition, sympathetic nerve blocks by local anesthesia may also lead to hypotension. Antihypertensive drugs such as calcium channel blockers, angiotensin II receptor antagonists, and alpha receptor blockers can aggravate the blood pressure reduction caused by local dental anesthesia (Ouchi & Jinnouchi, 2021).

During pregnancy, there is an increasing number of white blood cells, red blood cells, and blood clotting factors except for XI and XII, which leads to a fivefold increased likelihood of thromboembolism such as deep vein thrombosis and pulmonary embolism (Alsheef et al., 2020; Guimicheva, Czuprynska & Arya, 2015). Compression of the inferior vena cava in a supine position also contributes to venous stasis and thrombosis and increases the risk of deep vein thrombosis.

### Respiratory alterations

Progesterone can directly stimulate ventilation by increasing the sensitivity of the respiratory center to carbon dioxide, making gravid women require greater tidal volume to remove carbon dioxide (Costantine, 2014; Kohlhepp et al., 2018). Hyperventilation begins in the first trimester and may increase to 42% in the third trimester. Moreover, it has been reported that increased serum estrogen concentrations contribute to nasal capillary bleeding, which leads to nasal congestion and breathing difficulties (Costantine, 2014). Up to 50% of pregnant women complained of breathing difficulties at 19 weeks of gestation and up to 75% at 31 weeks (Lee et al., 2017; Tan & Tan, 2013).

In addition to the increased oxygen demands, there are concomitant decreases in oxygen reserves. To compensate for the space taken up by the fetus, the diaphragm moves up, and the residual lung volume drops by up to 20%, especially in the supine position. The mother and fetus are prone to hypoxia due to a decrease in oxygen reserves. Research has demonstrated that moderate hypoxemia occurs in 25% of pregnant women in the supine position. Even short periods of apnea can rapidly lead to maternal hypoxemia and hypercapnea (Zhao, Wong & Stevenson, 2021). Therefore, it is necessary to adjust the ventilation pattern and position to avoid hypoxemia.

### Gastrointestinal alterations

The main gastrointestinal changes during pregnancy are related to nausea, vomiting, and heartburn. Increased progesterone levels during pregnancy have been linked to decreased esophageal tone, delayed gastric emptying, and decreased bowel motility, which may lead to gastroesophageal reflux during pregnancy. A supine position can lead to decreased arterial partial oxygen pressure, increasing the risk of gastroesophageal reflux dyspepsia, which can cause aspiration of gastric contents (Costantine, 2014). Therefore, if the patient feels nauseous during the treatment, the operation should be stopped immediately, and the dental chair should be upright.

### Liver, renal and endocrine alterations

The production of estrogen and progesterone increases during pregnancy and reaches a maximum during the third trimester. These hormonal changes can affect liver function. The decreased synthesis capacity of the liver results in decreased levels of albumin and alpha-1 acid glycoprotein, leading to an increase in the proportion of uncombined drugs (Lim, Mouyis & MacKillop, 2021). The medications should have their doses reduced accordingly to the increased distribution and enhanced drug action (Avram, 2020).

Another typical change in pregnant women is an increased glomerular filtration rate (GFR) due to increased cardiac output (Bonnet, 2016; Habak & Griggs, 2022). As a result of the increased filtration capacity, the clearance of albumin, glucose, creatinine, and urea increased, as did the clearance of drugs. Drugs such as lidocaine, cleared by the kidneys, should have their doses adjusted to the clearance rates (Calimag-Loyola & Lerma, 2019; Uppal et al., 2022).

In addition, about 45% of pregnant women develop gestational diabetes during pregnancy (Chiou et al., 2022; Schwartz, Nachum & Green, 2015). Epinephrine injected during local oral anesthesia can directly increase circulating glucose levels, especially in patients with poor glycemic control (Byakodi, Gurjar & Soni, 2017). Therefore, detailed preoperative inquiry and adding epinephrine with caution are necessary.

The incidence of hypoglycemic syncope also increases during pregnancy. Glucose excretion increases in pregnant women due to increased GFR during pregnancy. In addition, estrogen and progesterone can increase the utilization of glucose. The pregnant woman’s increased metabolism and the fetus’s glucose uptake result in increased glucose requirements. Some pregnant women are also stressed when they feel pain or see dental instruments, which increases the secretion of gastric acid and pepsin in the digestive system and aggravates gastric peristalsis. Under the combined effect of the above factors, pregnant women have lower blood glucose and are susceptible to hypoglycemic syncope.

### Neuropsychological alterations

Adequate and effective psychological counseling is essential for pregnant women before the clinical operation to reduce their fear of treatment. The sensitivity of nerve fibers to local anesthetics during pregnancy is higher than that during non-pregnancy, so the onset time of conduction block is earlier. In addition to the improved anesthesia effect, mental tension and anxiety will be aggravated during pregnancy (Jarvis et al., 2012). Increased fear and sensitivity to pain can lead to increased heart rate and blood pressure, which can affect the stability of the fetus and even cause epileptic seizures and abortion (Benson & Pack, 2020; Whelehan & Delanty, 2019). Seizures are accompanied by increased catecholamine production, followed by decreased blood flow to the placenta. Tissue acidosis and hypoxia are common findings in systemic seizures, which decrease the plasma protein binding rate of local anesthetic. Once local anesthetics enter the bloodstream, toxic effects on the central nervous system further increase.

### Oral health alterations

Recent studies have shown an association between caries and pregnancy (Hu et al., 2022). Specifically, the need for carbohydrates during pregnancy often leads to hunger and increased eating times. Pregnant women inevitably experience vomiting and aggravated pharyngeal reflex, which brings more food residue. The stomach acid contained in this vomit also causes dental erosion. Acidic foods favored by pregnant women can also lower the pH of saliva. Changes in the composition of saliva can temporarily make teeth more prone to erosion and decay (Doucède et al., 2019). Moreover, increased estrogen levels in saliva lead to an increased desquamation rate of the oral mucosa, which increases bacterial proliferation and promotes tooth decay (Yousefi, Parvaie & Riahi, 2020). Pregnancy also weakens the immune system and affects and accelerates the progression of caries. Caries during pregnancy should be treated in time to avoid abortion and premature delivery caused by severe toothache.

Approximately 60% to 75% of pregnant women have gingivitis, especially in the third trimester of pregnancy (Vigarios & Maret, 2020). Due to the increase in estrogen and progesterone levels, insulin sensitivity decreases, and sugar metabolism changes during pregnancy. As a result of the increased blood sugar and volume, the minute vessel expands, elasticity decreases, and blood capillary permeability increases. During pregnancy, the inflammatory response is highly activated, neutrophil function is suppressed, and the expression of inflammatory markers is increased (Choi et al., 2021; Mahapatra et al., 2021; Tettamanti, 2017). Along with the increase in inflammatory cells and exudate, the gums are often congested and swollen with hypertrophic inflammation during pregnancy (González-Jaranay et al., 2017; Vigarios & Maret, 2020). Supposing there is a large amount of plaque and calculus accumulation in this period, gingival inflammation will be heightened and finally form periodontal disease. Periodontal treatment is encouraged to alleviate suffering and reduce the risk of premature birth and other adverse reactions during pregnancy (Iheozor-Ejiofor et al., 2017; Iqbal et al., 2022).

Another pathological gum state that occurs during pregnancy is pyogenic granuloma, with an incidence of about 3% to 10%. Lesions are similar to benign tumors caused by the growth of connective tissue in the gums and most often occur in areas of inflammation or chronic trauma in the gums (Sarwal & Lapumnuaypol, 2022; Silva de Araujo Figueiredo et al., 2017). Clinical cleaning and curettage are often used to remove local irritation in the affected area. Prompt surgical removal is required for large gingival tumors with persistent pain or infection (Guastella et al., 2017).

Third molar pericoronitis can cause not only affliction but also fever and fascial space infection. Patients have difficulty eating, chewing, and swallowing and restricted mouth opening, which can cause infection of adjacent tissues and organs or interstitial space in severe cases. The treatment choices for mild symptoms are local irrigation to control symptoms or removal under close supervision. In the case of interstitial infection, abscess incision and drainage are recommended under anesthesia.

Emergency oral treatment during pregnancy is often accompanied by local acute inflammation, which causes difficulty in anesthesia. Research has demonstrated that the success rate of lidocaine block anesthesia in patients with pulpitis is only 67%, much lower than that in normal teeth (Visconti, Tortamano & Buscariolo, 2016). Inflammation and bacteria can lead to increased expression of neuropeptides such as Substance P and calcitonin gene-related peptides, as well as the release of inflammatory mediators such as prostaglandin E2, prostaglandin F2α, and interleukin 1 and 6, leading to the excitement of pain receptor subtypes. Moreover, expression of the sodium channel increases in inflamed dental pulp, which results in the alteration of resting membrane potential, and the decreased excitability threshold of the teeth (Wang et al., 2021). Clinical manifestations include changes in neuronal plasticity, abnormal pain, and peripheral and central hyperalgesia (Drum et al., 2017). The acidic tissue environment of inflammation also makes it difficult for drugs to be converted into deionized forms of active free radicals, making it difficult for lidocaine molecules to cross nerve membranes, resulting in poor anesthesia effects (Yilmaz, Tunga & Ozyurek, 2018).

### Fetal considerations

Most drugs given to pregnant women may affect the fetus after they are transferred across the placenta and enter the systemic circulation of the fetus. The effects of the transferred drug on the fetus depend on the type of drug and the general fetal conditions (Bouazza et al., 2019; Kolding, Eken & Uldbjerg, 2020; van Hove et al., 2022). Therefore, it is essential to understand the pharmacology of local anesthetic, the most commonly used drugs in dental treatment, for safe and effective dental treatment during pregnancy (Ouanounou & Haas, 2016).

The severity of the effects of local anesthetic on the fetus depends on the dose, whether a vasoconstrictor is used, the metabolic rate of local anesthetic, the degree of protein binding, and pKa (acid dissociation constant). The degree of binding of local anesthetic to maternal protein has the most significant effect. Only unconjugated anesthetic molecules can be transferred across the placenta to the fetus (Pemathilaka, Reynolds & Hashemi, 2019). For the fetus, the potential risks associated with anesthesia are mainly fetal hypoxia, miscarriage or premature delivery, and to a lesser extent teratogenicity (Tolcher, Fisher & Clark, 2018; Vasco Ramirez & Valencia, 2020).

### Teratogenicity

“No currently utilized anesthetic drugs have been found to have any teratogenic effects in humans when utilizing standard concentrations at any gestational age,” claims the American College of Obstetricians and Gynecologists (2017). Multiple clinical studies have demonstrated that oral local anesthesia and oral treatment during pregnancy does not increase the risk of fetal malformation within the maximum safe dose (Hagai et al., 2015; Michalowicz et al., 2008; Moore, 2016). Herein, dentists who are cautious and reluctant to use local anesthetics on pregnant women should change their views and believe that women can receive necessary dental treatment during pregnancy as long as the anesthetic is chosen correctly and the dosage is controlled.

### Hypoxia and asphyxia

The most severe adverse fetal event of anesthesia during pregnancy is intrauterine asphyxia. Due to the lack of automatic regulation of placental perfusion and the complete dependence of fetal oxygenation on maternal oxygenation, any hemodynamic changes in the mother will directly affect uteroplacental perfusion. Prolonged or severe maternal hypotension, hypoxemia, and hypercapnia can result in decreased uterine placental blood flow and fetal ischemia (Lato et al., 2018). Injecting small amounts of local anesthetics into the head and neck region may also result in unfavorable effects that are comparable to systemic toxicity caused by inadvertent intravascular injections of higher dosages. Cardiovascular stimulation or depression, disorientation, seizures, and respiratory depression have been reported. These reactions could be the result of intra-arterial local anesthetic injections with retrograde cerebral circulatory flow (Johnson, Boscoe & Cabrera-Muffly, 2020). Epinephrine can reduce blood flow in the uterus and uterine contractile force proportionally to the dose when given intravenously (Hood, Dewan & James, 1986), further inducing fetal hypoxia (Badran et al., 2019; Ritchie et al., 2017). Although there have been no cases of oral local anesthetic causing fetal hypoxia, oral local anesthetic may do so in expectant mothers (Tomlin, 1974). Whilst under block anesthetic, patients should have their breathing and circulation closely monitored. Dose guidelines shouldn’t be surpassed. In anoxic fetuses, the binding of the local anesthetic to the protein is reduced compared to healthy fetuses, and lidocaine is retained due to tissue acidosis (Ralston & Shnider, 1978). Thus, fetal sensitivity to neurotoxicity and cardiovascular toxicity of local anesthetic increases. Therefore, extra attention should be given to maintaining maternal blood pressure and oxygenation. Aspiration should be done before to injecting the local anesthetic solution in order to prevent intravascular injection. The needle must be adjusted until aspiration produces no return of blood. For fetuses at high risk of asphyxia or in poor general condition, local anesthetic and epinephrine must be used with caution (Bonnet, 2016).

### Miscarriage and premature birth

Sporadic spontaneous abortion occurs most often in the first trimester of pregnancy. Premature delivery is considered under 37 weeks (Purisch & Gyamfi-Bannerman, 2017). Although local anesthesia has no observed adverse impact on healthy fetuses, the risk is higher for pregnant women with medical conditions such as heart disease and pre-eclampsia. Pre-eclampsia is a pregnancy complication characterized by high blood pressure, proteinuria, and edema in 3–7% of pregnant women (Rana et al., 2019). On the one hand, there is a maternal syndrome characterized by endothelial cell activation, blood pressure disturbance, proteinuria, and edema; on the other hand, there is reduced intrauterine growth of the fetus (Phipps et al., 2016; Rana et al., 2019). Pre-eclampsia in pregnant women has reduced protein binding to local anesthetic. When placental perfusion is suddenly reduced, many free forms of local anesthetic can be transferred to the fetus, resulting in premature delivery.

Therefore, the type and dosage of local anesthetic must be carefully considered for pregnant women with underlying health problems. The management of anesthesia in pregnant women should focus on the hemodynamic stability of the mother to avoid hypoxemia and alkalosis or acidosis (Ralston & Shnider, 1978). Abdominal pain during the procedure may indicate premature labor or miscarriage. If this occurs, dentists should stop the operation immediately and call the obstetrician for help (Vasco Ramirez & Valencia, 2020).

## Preoperative prophylaxis

### The timing of the operation

Currently, it is not recommended to postpone dental treatment during pregnancy (educational, preventive, and restorative actions), while significant traumatic periodontal surgery should be avoided during pregnancy. The best time for dental treatment is between 14 and 20 weeks of pregnancy (OHRC, 2023). The fundamental reason for this approach is that pregnant women will experience considerable psychological and physiological changes that they have not fully adapted to during early pregnancy. In addition, many pregnant women experience severe morning sickness during the first trimester, which complicates clinical therapy (CDA, 2010). In the third trimester of pregnancy, the enlarged uterus makes it difficult to lie in the chair for a long time, which increases the difficulty of complex oral treatment. The supine position is more prone to inferior aortic vena cava compression and lead to postural hypotension, which needs to be alleviated by placing the pregnant woman in a semi-decumbent position and changing the position frequently. The recommendation for the second trimester is based on psychological and physical comfort and does not imply that oral therapy should be prohibited in the first and third trimesters. Prompt treatment of oral diseases and infections is beneficial to women at all stages of pregnancy. Pregnancy should not be a reason to delay treatment for oral emergencies at any point in pregnancy, as acute dental pain or infection can have serious adverse consequences for the pregnant woman and fetus (Kapila, 2021).

### Preoperative assessment of physical and psychological status

The combination of fear of LA injection and oral therapy can cause severe psychological discomfort and sometimes potentially life-threatening conditions, such as vascular suppression syncope, hyperventilation, clonic convulsions, bronchospasm, angina pectoris, *etc*. (Armfield & Heaton, 2013). Physicians and dentists should inquire about medical history and pregnancy-related causes in detail, paying particular attention to any conditions that raise the patient’s blood pressure.

### Local anesthesia agents

To determine the risks associated with drugs used during pregnancy, the United States Food and Drug Administration (FDA, 1979) classified drugs according to their risk level to the fetus (Table 1). Categories A and B are considered safe with no adverse effects on the fetus. Drugs in category C have been shown to have teratogenic risk in the animal fetus, but there is no adequate evidence in humans (Lee & Shin, 2017). Lidocaine and prilocaine are rated B by the FDA and are considered the safest local anesthetic for pregnant women. Because the concentration of prilocaine (4%) is higher than that of lidocaine (2%), resulting in more drugs being administered per injection, lidocaine is the preferred choice in the clinic.

**Table 1:** Summary of local anesthetics used in pregnant dental patients (FDA, 1979).

Agent	FDA category	Using during pregnancy
Articaine	C	Use with caution
Bupivacaine	C	Use with caution
Benzocaine	C	Use with caution
Mepivacaine	C	Use with caution
Lidocaine	B	Safe
Prilocaine	B	Safe

Another commonly used local anesthetic, articaine, is in category C (FDA, 2023). The combination of articaine hydrochloride and epinephrine has been proven to increase fetal mortality and skeletal variation in rabbits when administered subcutaneously at four times the maximum recommended dose (FDA). The FDA recommends that articaine hydrochloride and epinephrine should only be used during pregnancy if the potential benefits outweigh the potential risks to the fetus.

### Pharmacological characteristics of lidocaine

After injection, lidocaine is buffered by body fluids and dissociated into an uncharged lipid-soluble base and a relatively lipid-insoluble cation in equilibrium. The base form penetrates the nerve membrane into the nerve axon. The amount of base type is closely related to the pKa of local anesthetic and the pH value of tissues, according to the Henderson–Hasselbalch equation, pH = pKa + log(base/acid). After entering the nerve membrane, the free base form is converted back to the cation form, binds to the sodium channel, reduces the membrane’s permeability to sodium ions, and induces a non-depolarized nerve block (Malamed, 2014).

The liver metabolizes 90% of lidocaine, and 10% is excreted in its original form (FDA, 2021). Because lidocaine hydrochloride is metabolized quickly and can dilate blood vessels, most drugs are absorbed into the body quickly. This condition not only shortens the duration of anesthesia but also increases the risk of intoxication. Therefore, adrenaline is often added to lidocaine solutions to extend the duration and reduce the risk of poisoning (Ogawa et al., 2021).

### The epinephrine controversy

FDA classifies epinephrine as Pregnancy Category C because it is teratogenic in rabbits, mice, and hamsters dosed during organogenesis. Epinephrine should be used during pregnancy only if the potential benefit justifies the potential risk to the fetus (fetal anoxia, spontaneous abortion, or both) (FDA, 2022). Although epinephrine is injected outside the blood vessel, it has been shown to quickly enter the circulatory system after oral injection, with a peak occurring about 8 min after injection (Takahashi et al., 2005). Epinephrine is influential in various systemic diseases such as hyperthyroidism, glaucoma, and diabetes (FDA, 2022). Epinephrine, on the other hand, can significantly constrict blood vessels in the uterus and reduce blood flow to the placenta. In pregnant women with peripheral arterial obstructive and hypertensive vascular disease, epinephrine may exhibit a significant vasoconstriction response leading to ischemic injury or necrosis and must be used with caution. The increased risk may be even more significant in patients with severe cardiovascular disease or those taking medications that interact with epinephrine (FDA, 2022).

Most current studies considered that using epinephrine for LA during pregnancy is safe as long as the potential risks of intravascular injection are minimized and safe injection doses and methods are guaranteed (Ilyas et al., 2020). Clinical studies have proven that low-dose epinephrine used in dentistry does not significantly affect arrhythmia incidence, mean arterial pressure, heart rate, or other indicators, even in patients with cardiovascular disease (Guimaraes et al., 2021; Tschopp et al., 2018).

The improvement of the anesthesia effect with the addition of epinephrine is significant. One advantage is local vasoconstriction, resulting in a prolonged duration of anesthesia (Tschopp et al., 2018), reduced bleeding at the site of administration, and the increased concentration of local anesthetic in pulp tissue (Fujita & Sunada, 2021; Tanaka et al., 2016). The addition of epinephrine also delayed the absorption of the local anesthetic, resulting in a gradual increase in lidocaine levels in the blood with no peak and a peaceful transfer to the fetus with increased safety (Lee & Shin, 2017).

The concentration of epinephrine added to 2% lidocaine is still controversial, and the dosage form of 1:80,000–1:200,000 epinephrine is more commonly used clinically (Singla et al., 2022). Higher concentrations, such as 1:50,000, do not provide faster onset or longer duration for local oral anesthesia (Dagher, Yared & Machtou, 1997; Wali et al., 2010). Although high epinephrine concentrations can help reduce bleeding at the site of local invasion, there is a greater risk of acute epinephrine reactions such as hypertension and tachycardia (Giovannitti, Rosenberg & Phero, 2013). According to currently limited studies, it is safer to inject 2% lidocaine with 1:200,000 epinephrine. Although the anesthesia effect is slightly lower than that of the injections of 2% lidocaine with 1:80,000 epinephrine, there is no significant difference (Aggarwal et al., 2020; Karm et al., 2017, 2018).

To minimize the cardiovascular risks associated with epinephrine, clonidine, a hypertensive drug that can be used in pregnant women, may be an effective and safe alternative to epinephrine for LA during pregnancy. Multiple studies have shown that clonidine improves the success rate of lower alveolar nerve block anesthesia in patients with symptomatic irreversible pulpitis (MacDonald et al., 2021; Shadmehr et al., 2017), reduces postoperative pain and analgesic use (Shadmehr et al., 2017, 2021), and, most importantly, reduces cardiovascular risk compared with epinephrine (Chowdhury, Singh & Shah, 2012; Jimson et al., 2015; Patil & Patil, 2012).

## Intraoperative prophylaxis

### Improved anesthesia techniques

Discomfort and pain from LA injection may rapidly release large amounts of catecholamine, causing severe adverse reactions. Topical anesthesia before injection can eliminate or minimize pain caused by the needle, and undoubtedly add to the safety of LA in pregnant patients. Topical anesthesia and local cooling are commonly used to achieve surface tissue anesthesia in clinics (Maia et al., 2022; Patel et al., 2021). The commonly used topical anesthetics in the clinic include 8% lidocaine gel, 5% EMLA (2.5% lidocaine and 2.5% prilocaine) cream, and 20% benzocaine, among which only lidocaine and prilocaine are rated as B by the FDA and can be used during pregnancy. Moreover, precooling the injection site utilizing popsicle sticks, frozen cotton swabs, and ice packs is a simple, reliable, low-cost, and practical clinical method to reduce patient suffering (Amrollahi, Rastghalam & Faghihian, 2021). The analgesic effect is similar to that of 5% lidocaine gel, while the unpleasant taste of the gel is avoided (Hindocha et al., 2019). Vasoconstriction induced by precooling reduces tissue metabolism and the inflow of inflammatory mediators during needle penetration (Taylor et al., 2019). In addition, ice massage also stimulates A-δ fibers and activates inhibitory pain pathways, thereby delaying or eliminating pain signal transmission and raising pain thresholds (Anantharaj et al., 2020; Hameed et al., 2018; Jayasuriya, Weerapperuma & Amarasinghe, 2017; Lakshmanan & Ravindran, 2021).

Local infiltrating anesthesia is the primary injection method for maxillary anesthesia because of its high success rate and low risk of injection into blood vessels (Sandilya et al., 2019; Wang et al., 2021). However, the mucosa of the palate is close to the periosteum, and there is an extensive neural network, so infiltration anesthesia in the palatal region is painful and irritating. Multiple studies have shown that although the efficacy of buccal infiltration anesthesia of 2% lidocaine with epinephrine for maxillary molar extraction is still controversial, it can provide similar effects to buccal-palatal infiltration anesthesia in other maxillary dental regions (Deshpande et al., 2020; Phyo et al., 2020; Rayati et al., 2021). The effect of 2% lidocaine infiltration on the mandible is not sufficiently stable. Although lidocaine infiltration anesthesia has been reported to be sufficient for the extraction of mandibular incisors and premolars (Ege & Demirkol, 2021; Jamil, Asmael & Al-Jarsha, 2020), anesthesia for mandibular molars has a lower success rate or needs to be combined with other injection methods (Gazal et al., 2020). Attention should also be paid to the location of the pinhole, and the injection point should be as close to the nerve foramen as possible (Wang et al., 2021).

Nerve block anesthesia has incomparable advantages such as less anesthetic dosage, wider anesthesia range, and longer effective anesthesia time, and is especially suitable for a long time and a wide range of surgeries, such as mandibular third molar extraction. The downside is that the failure rate of inferior alveolar nerve block anesthesia (IANB) is up to 20–47% in symptomatic patients with irreversible pulpitis (Crowley et al., 2018). If routine IANB fails, patients with irreversible pulpitis often need supplementary injections such as intra-osseous anesthesia (Yadav, 2015), periodontal injection (Gupta et al., 2022; Wadia, 2022), and infiltrative anesthesia (Gazal et al., 2020; Gupta et al., 2021, 2022) to achieve complete anesthesia. In addition to conventional nerve block anesthesia, Goe-Gates effectively improved the success of IANB by moving the injection point up to the neck of the condyle (Aggarwal, Singla & Kabi, 2010). At the same time, the positive rate of blood recovery can be reduced to approximately 2% (Sokhov, Rabinovich & Bogaevskaya, 2019).

### New local anesthesia formulations

Topical anesthesia offers the possibility of painless dental anesthesia, but there are currently no commercially available formulations that eliminate the pain of local anesthesia injections (Hindocha et al., 2019). In order to enhance the permeability of lidocaine and improve the effectiveness of surface anesthesia, some materials, such as liposomes (Franz-Montan et al., 2015; Ribeiro et al., 2016), silica nanoparticles (Nafisi et al., 2018; Sato et al., 2019), and chitosan (Qi et al., 2021), have been gradually developed as lidocaine delivery systems. However, most lidocaine delivery systems focus on the transdermal route. Only a few studies have reported using the lidocaine liposome complex in the oral mucosa.

Ribeiro et al. (2016) evaluated the package formulation of the most effective lipid carrier for delivering 2.5% lidocaine and 2.5% prilocaine based on a factored design data analysis program. The prepared eutectic mixture loaded with pectin and liposomes has the advantages of long drug release time, high permeability, and long anesthesia time. Subsequently, yellow collagen was combined with the lipid carrier mentioned above to improve the adhesion of oral mucosa (Ribeiro et al., 2018). Cordeiro Lima Fernandes et al. (2021) also prepared a kind of prilocaine-lidocaine lipid nanogel, which also had the advantages of small particle size, good dispersion, and adhesion to oral mucosa and could effectively prolong the anesthesia time. A clinical study has also demonstrated that the lidocaine-prilocaine liposome complex can provide adequate palatal mucosal topical anesthesia for maxillary molar extraction without mucosal discomfort, allowing the physician to obtain complete anesthesia for approximately 26 min without injection (Amorim et al., 2020).

In addition to the combination with prilocaine, lidocaine oral mucosal patches can enhance surface anesthesia efficacy. Roh et al. (2016) developed an oral adhesive bilayer containing lidocaine that enabled 80% of lidocaine to be released within 1 min (*p* < 0.05), effectively permeate the mucosa (*p* < 0.05), and remain attached for at least 3 h. Microneedle patches applied to the oral mucosa before lidocaine injection are also reported to be effective in reducing the pain of injection (Daly et al., 2021). 3D printing has been used to create a customizable lidocaine-loaded patch that perfectly attaches to the tooth for more than 1 h while releasing lidocaine from the hydrogel (Ou et al., 2019).

### Injection dose

Pregnant patients have a higher risk of suffering from local anesthesia intoxication due to complex physiological changes, making the control of anesthetic dose particularly crucial. Fortunately, clinical studies have shown that using local dental anesthesia during pregnancy does not increase the risk of fetal malformation at normal dental doses (Fisher et al., 2020; Hagai et al., 2015; Moore, 2016). Therefore, dentists who are cautious and reluctant to use local anesthesia on pregnant women should revise their views about dental treatment for pregnant women. Women can receive necessary dental treatment during pregnancy if they choose the proper anesthetic and control the dosage correctly.

The American Dental Association Dental Treatment Guidelines state that dentists should use the lowest concentration and volume of anesthetic fluid that provides adequate anesthesia (2009). The maximum dosage of lidocaine may be calculated to be 4.5 mg/kg, and the total dose should be at most 300 mg. The dose of lidocaine in combination with epinephrine should not exceed 7 mg/kg, and the maximum total dose should not exceed 500 mg (FDA, 2021). Moreover, the maximum dosage of epinephrine when used with local anesthetic is less than 0.2 mg for healthy patients and less than 0.04 mg for patients with heart disease (FDA, 2022; Fleisher Lee et al., 2014). The maximal dose of lidocaine for local anesthetic is presented in Table 2 (FDA, 2021; Fleisher Lee et al., 2014).

**Table 2:** The maximum recommended doses of lidocaine (FDA, 2021; Fleisher Lee et al., 2014).

Volume of cartridge	Agents	Concentration		Maximum dosing			Maximum number of cartridges		
		Lidocaine (mg/cartridge)	Epi (mg/cartridge)	Lidocaine (mg)	Lidocaine (mg/kg)	Epi (mg)	50 kg patient	65 kg patient	80 kg patient
1.8 ml	2% lidocaine, 1:100,000 epi	36	0.018	500	7	0.2	9	11	11
	2% lidocaine, 1:80,000 epi	36	0.0225	500	7	0.2	8	8	8
	2% lidocaine, 1:50,001 epi	36	0.036	500	7	0.2	5	5	5
	2% lidocaine, plain	36	–	300	4.5	–	6	8	8
5 ml	2% lidocaine, 1:100,000 epi	100	0.05	500	7	0.2	3	4	4
	2% lidocaine, 1:80,000 epi	100	0.0625	500	7	0.2	3	3	3
	2% lidocaine, 1:50,000 epi	100	0.1	500	7	0.2	2	2	2
	2% lidocaine, plain	100	–	300	4.5	–	2	2	3

**Note:**

Abbreviations: epi, epinephrine.

## How to minimize injection pain

### Buffering

**Figure 2:**
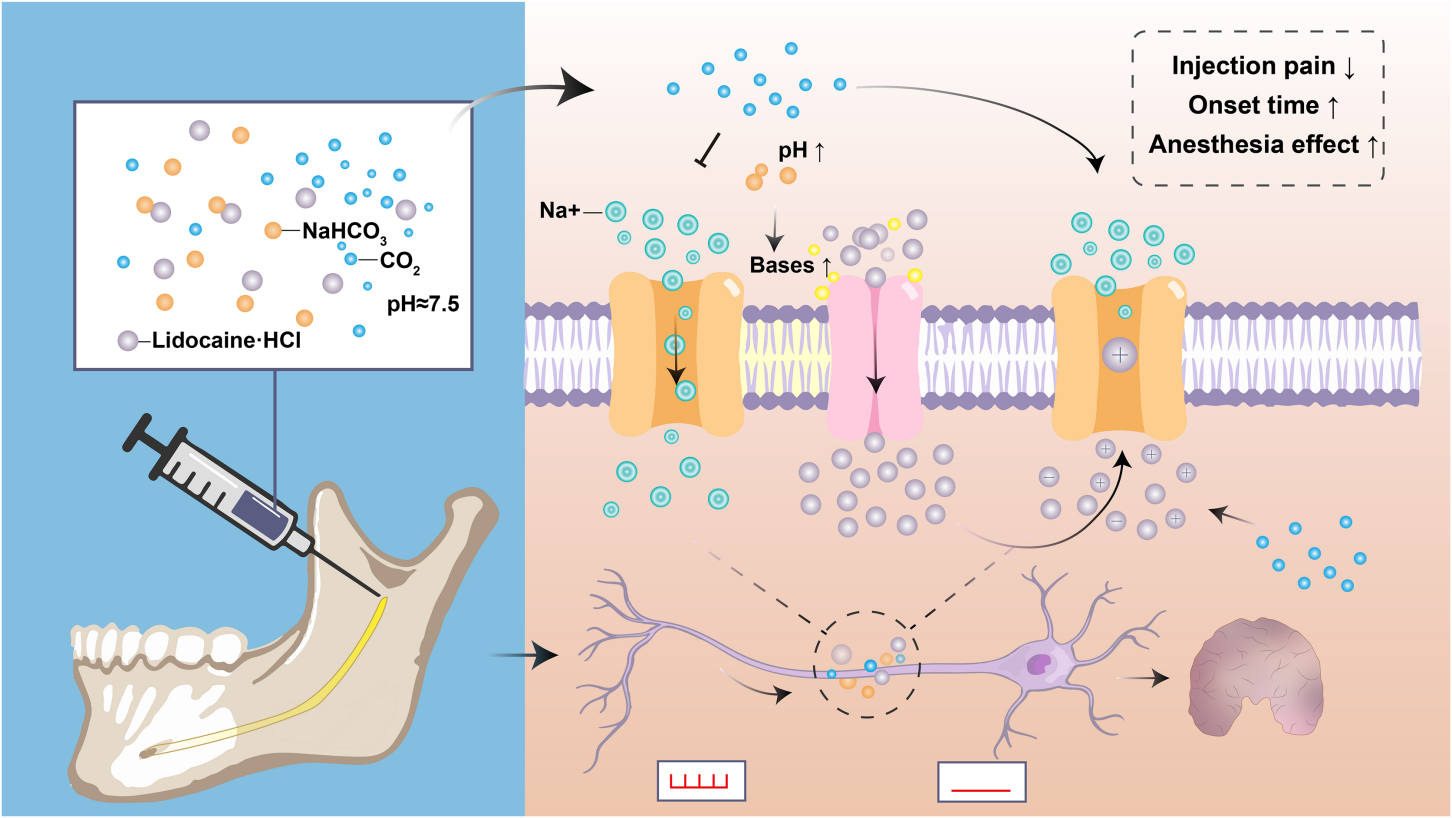
Diagram depicting the applications and mechanisms of sodium bicarbonate buffering. Sodium bicarbonate buffering raises anesthesia pH, and the rapid formation of a mixture of charged and uncharged forms leads to more rapid drug diffusion and faster onset of nerve block, and makes injections more comfortable. Carbon dioxide as a byproduct may enhance anesthesia by directly inhibiting nerve conduction and by concentrating local anesthetic through ion retention.

As described above, the molecular and dissociated lidocaine ratio is determined by the anesthetic’s pH and pKa and the tissue’s pH. The pH of most lidocaine preparations is reduced to 3–4 by hydrochloric acid because the charged form of the lidocaine molecule is more stable at low pH (Malamed, 2014). However, the acidification of lidocaine has increased the sting on injection and the onset of deep anesthesia, and reduced the anesthesia effect of numbing inflamed or infected teeth (Malamed, 2014; Yilmaz, Tunga & Ozyurek, 2018). Theoretically, the ability of anesthetic molecules to penetrate the nerve membrane depends on base concentration, which is enhanced when the increase in the number of bases at high pH allows more anesthetic to exist in their non-ionizing form. The active form of free radicals penetrating the nerve membrane into the inner part of the neuron is increased, thus shortening the onset time and reducing the pain of injection (Amorim et al., 2021; Arora, Degala & Dasukil, 2019). Additionally, when the sodium bicarbonate solution is mixed with lidocaine, the sodium bicarbonate interacts with hydrochloric acid in the anesthetic to produce water and carbon dioxide. Carbon dioxide can enhance local anesthesia by directly inhibiting the nerve axons, increasing the concentration of lidocaine in the nerve stem, and changing the charge of local anesthesia in the nerve (Fig. 2) (Catchlove, 1972).

Multiple clinical trials have also supported the theory that sodium bicarbonate reduces the pain of injection and improves the anesthesia effect by buffering lidocaine (Kattan et al., 2019; Kurien, Goswami & Singh, 2018). Interestingly, a study of 96 patients with periapical infections requiring tooth extraction showed that a single injection of 0.5 ml 8.4% sodium bicarbonate significantly relieved pain after lidocaine anesthesia failed (*p* < 0.1) (Senthoor, Janani & Ravindran, 2020). However, there have also been clinical studies showing different results (Aulestia-Viera, Braga & Borsatti, 2018; Guo et al., 2018). Since the therapeutic use of sodium bicarbonate as an alkalizing agent for local anesthetic has not been approved, it should be used with caution.

### Warming

The pKa value of local anesthetic will decrease with increasing temperature. Under the condition in constant pH, the decrease of pKa will increase the proportion of free radicals from drugs, and thus increase their passive diffusion in non-neural structures, helping to block the transmission of pain signals (Ince et al., 2021; Lim et al., 1992). In addition, increased temperature also increases the fluidity of the lipid membrane, allowing lidocaine to penetrate the membrane and reach effective concentrations for faster analgesia. The enhancement of the anesthesia effect may also be related to the transient receptor potential vanilloid (TRPV1) channel in trigeminal nerve tissue, which is activated in near-harmful temperature ranges (over 42 °C). Once activated, the widened membrane gap facilitates the infiltration of cations, thereby increasing the concentration of lidocaine molecules within the nerve cells (Binshtok et al., 2009; Caterina et al., 1997; Zhou et al., 2020).

Clinical results have also demonstrated that the use of warmed lidocaine for inferior alveolar nerve block anesthesia results in a faster onset of anesthesia (*p* = 0.004) and less pain (*p* < 0.001) in patients (Kurien, Goswami & Singh, 2018). Tirupathi & Rajasekhar (2020) included four studies from 2018 to 2020 to evaluate the subjective and observed pain reactions and found that heating the local anesthesia solution to body temperature (37 °C) before administration reduced discomfort during oral local anesthesia administration.

Moreover, a significant source of injection pain is subcutaneous tissue expansion. Therefore, slow injection has a significant effect in reducing pain (Garret-Bernardin et al., 2017). When used for oral treatment under local anesthesia, lidocaine should be injected at less than 1 ml/min (FDA, 2021; Tangen et al., 2016). In addition, distracting methods such as kneading the patient’s cheeks have been proven to relieve pain during anesthesia injections (Birnie et al., 2018; Chen et al., 2020).

### Applicable syringes

Currently, many innovative dental syringes, such as computer-controlled autoinjectors, are gradually being applied clinically to reduce the discomfort of standard local anesthesia. The vibration technique was first applied to reduce pain. The impression of “pain” from the pressure of the fluid entering the tissue is lessened when the brain activity is concentrated on the vibration (Nanitsos et al., 2009). Vibration and touch also stimulate inhibitory interneurons in the spinal cord, which transmit information to second-order neurons in the spinal cord *via* the A-δ and C fibers, and ultimately lead to pain elimination (Dickenson, 2002). Computer-aided devices, such as VibraJect, Dental Vibe, Accupal, and Wand, can significantly reduce pain at the injection site, relying on machine vibrations (Bilsin, Güngörmüş & Güngörmüş, 2020; Joshi et al., 2021; Midha et al., 2021; Salma et al., 2021).

Intraosseous anesthesia is the injection directly into the cancellous bone near the tooth to be anesthetized. It can achieve deep anesthesia without numbness of the lip and cheek tissue and is mainly used in root canal practice. Intraosseous anesthesia can significantly enhance the effect of pulp anesthesia in patients with irreversible pulpitis and can be used for supplementary anesthesia after the failure of IANB alone (Dias-Junior et al., 2021; Kc, Bhattarai & Subedi, 2022; Zanjir et al., 2019). The continuous improvement of intraosseous anesthesia equipment is receiving increasing attention due to its good anesthesia effect, minimal invasiveness, simple operation, and easy control (Sovatdy et al., 2018).

## Conclusions

Although pregnancy is a particular event characterized by systemic alterations, it is necessary and safe to treat oral diseases that require treatment. Local anesthetics remain the safest and most efficient agents in dentistry to relieve intraoperative and postoperative pain. Local anesthesia is safe for both mother and fetus as long as a safe anesthetic is chosen and detailed guidelines are followed. Emerging injection techniques and anesthetic formulations have proven effective in a more extensive age range. Nevertheless, pain is a multi-factorial problem. It is still necessary to evaluate the effect of the new technology on pregnant women before clinical promotion. Joint efforts should be made to develop an obstetrician-dental document, which is necessary to remove doubt, uncertainty, or unnecessary suffering.

## Additional Information and Declarations

### Data Availability

The following information was supplied regarding data availability: This is a literature review.

## References

[ref-1] AAPD (2023). The reference manual of pediatric dentistry. https://www.aapd.org/research/oral-health-policies--recommendations/oral-health-care-for-the-pregnant-adolescent.

[ref-2] ADA (2021). Pregnancy. https://www.ada.org/resources/research/science-and-research-institute/oral-health-topics/pregnancy.

[ref-4] Aggarwal V, Singla M, Kabi D (2010). Comparative evaluation of anesthetic efficacy of Gow-Gates mandibular conduction anesthesia, Vazirani-Akinosi technique, buccal-plus-lingual infiltrations, and conventional inferior alveolar nerve anesthesia in patients with irreversible pulpitis. Oral Surgery, Oral Medicine, Oral Pathology, Oral Radiology, and Endodontology.

[ref-5] Aggarwal V, Singla M, Saatchi M, Hasija M (2020). Anaesthetic efficacy of 2% lidocaine with different concentrations of epinephrine (1:80,000 and 1:200,000) in intraligamentary injection after a failed primary inferior alveolar nerve block: a randomized double-blind study. Acta Odontologica Scandinavica.

[ref-6] Al Khamis S, Asimakopoulou K, Newton JT, Daly B (2016). Oral health knowledge, attitudes, and perceptions of pregnant kuwaiti women: a qualitative study. JDR Clinical & Translational Research.

[ref-7] Aleksenko L, Quaye IK (2020). Pregnancy-induced cardiovascular pathologies: importance of structural components and lipids. American Journal of the Medical Sciences.

[ref-8] Alsheef MA, Alabbad AM, Albassam RA, Alarfaj RM, Zaidi ARZ, Al-Arfaj O, Abu-Shaheen A (2020). Pregnancy and venous thromboembolism: risk factors, trends, management, and mortality. BioMed Research International.

[ref-9] American College of Obstetricians and Gynecologists (2017). Committee opinion No. 696: nonobstetric surgery during pregnancy. Obstetrics and Gynecology.

[ref-10] Amorim KS, Fontes VTS, Gercina AC, Groppo FC, Souza LMA (2021). Buffered 2% articaine versus non-buffered 4% articaine in maxillary infiltration: randomized clinical trial. Clinical Oral Investigations.

[ref-11] Amorim KS, Franz-Montan M, Groppo FC, Muniz BV, Araújo JSM, Santana JVF, Dantas A, de Paula E, Souza LMA (2020). Palatal needle-free anesthesia for upper molars extraction. A randomized clinical trial. Journal of Cranio-Maxillofacial Surgery.

[ref-12] Amrollahi N, Rastghalam N, Faghihian R (2021). Effect of pre-cooling on pain associated with dental injections in children: a systematic review. Journal of Evidence Based Dental Practice.

[ref-13] Anantharaj A, Sabu JM, Ramakrishna S, Jagdeesh RB, Praveen P, Shankarappa PR (2020). A comparative evaluation of pain perception following topical application of benzocaine gel, clove-papaya based anesthetic gel and precooling of the injection site before intraoral injections in children. Journal of Indian Society of Pedodontics and Preventive Dentistry.

[ref-14] Armfield JM, Heaton LJ (2013). Management of fear and anxiety in the dental clinic: a review. Australian Dental Journal.

[ref-15] Arora G, Degala S, Dasukil S (2019). Efficacy of buffered local anaesthetics in head and neck infections. British Journal of Oral and Maxillofacial Surgery.

[ref-16] Aulestia-Viera PV, Braga MM, Borsatti MA (2018). The effect of adjusting the pH of local anaesthetics in dentistry: a systematic review and meta-analysis. International Endodontic Journal.

[ref-17] Avram M (2020). Pharmacokinetic studies in pregnancy. Seminars in Perinatology.

[ref-18] Badran M, Abuyassin B, Ayas N, Laher I (2019). Intermittent hypoxia impairs uterine artery function in pregnant mice. The Journal of Physiology.

[ref-19] Barr LC, Liblik K, Johri AM, Smith GN (2022). Maternal cardiovascular function following a pregnancy complicated by preeclampsia. American Journal of Perinatology.

[ref-20] Benson R, Pack A (2020). Epilepsy. Handbook of Clinical Neurology.

[ref-21] Bilsin E, Güngörmüş Z, Güngörmüş M (2020). The efficacy of external cooling and vibration on decreasing the pain of local anesthesia injections during dental treatment in children: a randomized controlled study. Journal of Perianesthesia Nursing.

[ref-22] Binshtok AM, Gerner P, Oh SB, Puopolo M, Suzuki S, Roberson DP, Herbert T, Wang CF, Kim D, Chung G, Mitani AA, Wang GK, Bean BP, Woolf CJ (2009). Coapplication of lidocaine and the permanently charged sodium channel blocker QX-314 produces a long-lasting nociceptive blockade in rodents. Anesthesiology.

[ref-23] Birnie KA, Noel M, Chambers CT, Uman LS, Parker JA (2018). Psychological interventions for needle-related procedural pain and distress in children and adolescents. Cochrane Database of Systematic Reviews.

[ref-24] Bobetsis YA, Graziani F, Gürsoy M, Madianos PN (2020). Periodontal disease and adverse pregnancy outcomes. Periodontology 2000.

[ref-25] Bonnet M (2016). Sedation and anaesthesia for non-obstetric surgery. Anaesthesia Critical Care & Pain Medicine.

[ref-26] Bouazza N, Foissac F, Hirt D, Urien S, Benaboud S, Lui G, Treluyer JM (2019). Methodological approaches to evaluate fetal drug exposure. Current Pharmaceutical Design.

[ref-27] Byakodi S, Gurjar V, Soni S (2017). Glucose levels and hemodynamic changes in patients submitted to routine dental extraction under local anesthesia with and without adrenaline. The Journal of Contemporary Dental Practice.

[ref-28] Calimag-Loyola A, Lerma E (2019). Renal complications during pregnancy: in the hypertension spectrum. Disease-a-Month.

[ref-29] Catchlove R (1972). The influence of CO_2_ and pH on local anesthetic action. Journal of Pharmacology & Experimental Therapeutics.

[ref-30] Caterina MJ, Schumacher MA, Tominaga M, Rosen TA, Levine JD, Julius D (1997). The capsaicin receptor: a heat-activated ion channel in the pain pathway. Nature.

[ref-31] CDA (2010). Oral health during pregnancy and early childhood: evidence-based guidelines for health professionals. Journal of the California Dental Association.

[ref-32] Chen YJ, Cheng SF, Lee PC, Lai CH, Hou IC, Chen CW (2020). Distraction using virtual reality for children during intravenous injections in an emergency department: a randomised trial. Journal of Clinical Nursing.

[ref-33] Chiou Y, Hung C, Yu C, Chan T, Liu M (2022). Risk factors for women with gestational diabetes mellitus developing type 2 diabetes and the impact on children’s health. Journal of Clinical Nursing.

[ref-34] Cho GJ, Kim SY, Lee HC, Kim HY, Lee KM, Han SW, Oh MJ (2020). Association between dental caries and adverse pregnancy outcomes. Scientific Reports.

[ref-35] Choi S, Choudhary A, Ahern J, Palmer N, Barrow J (2021). Association between maternal periodontal disease and adverse pregnancy outcomes: an analysis of claims data. Family Practice.

[ref-36] Chowdhury S, Singh M, Shah A (2012). Efficacy of lignocaine with clonidine and adrenaline in minor oral surgical procedure. Contemporary Clinical Dentistry.

[ref-37] Cordeiro Lima Fernandes P, David de Moura L, Freitas de Lima F, Henrique Rodrigues da Silva G, Isaias Carvalho Souza R, de Paula E (2021). Lipid nanocapsules loaded with prilocaine and lidocaine and incorporated in gel for topical application. International Journal of Pharmaceutics.

[ref-38] Costantine MM (2014). Physiologic and pharmacokinetic changes in pregnancy. Frontiers in Pharmacology.

[ref-39] Crowley C, Drum M, Reader A, Nusstein J, Fowler S, Beck M (2018). Anesthetic efficacy of supine and upright positions for the inferior alveolar nerve block: a prospective, randomized study. Journal of Endodontics.

[ref-40] Dagher FB, Yared GM, Machtou P (1997). An evaluation of 2% lidocaine with different concentrations of epinephrine for inferior alveolar nerve block. Journal of Endodontics.

[ref-41] Daly S, Claydon NCA, Newcombe RG, Seong J, Addy M, West NX (2021). Randomised controlled trial of a microneedle patch with a topical anaesthetic for relieving the pain of dental injections. Journal of Dentistry.

[ref-42] Deshpande N, Jadhav A, Bhola N, Gupta M (2020). Anesthetic efficacy and safety of 2% lidocaine hydrochloride with 1:100,000 adrenaline and 4% articaine hydrochloride with 1:100,000 adrenaline as a single buccal injection in the extraction of maxillary premolars for orthodontic purposes. Journal of Dental Anesthesia and Pain Medicine.

[ref-43] Dias-Junior LCL, Bezerra AP, Schuldt DPV, Kuntze MM, de Luca Canto G, da Fonseca Roberti Garcia L, da Silveira Teixeira C, Bortoluzzi EA (2021). Effectiveness of different anesthetic methods for mandibular posterior teeth with symptomatic irreversible pulpitis: a systematic review and meta-analysis. Clinical Oral Investigations.

[ref-44] Dickenson AH (2002). Gate control theory of pain stands the test of time. British Journal of Anaesthesia.

[ref-45] Doucède G, Dehaynin-Toulet E, Kacet L, Jollant B, Tholliez S, Deruelle P, Subtil D (2019). Tooth and pregnancy, a public health issue. La Presse Médicale.

[ref-46] Drum M, Reader A, Nusstein J, Fowler S (2017). Successful pulpal anesthesia for symptomatic irreversible pulpitis. Journal of the American Dental Association.

[ref-47] Ege B, Demirkol M (2021). Is the only buccal infiltration anesthesia enough for extraction of mandibular anterior incisors and premolar teeth? A split-mouth randomized clinical trial. Clinical Oral Investigations.

[ref-48] Favero V, Bacci C, Volpato A, Bandiera M, Favero L, Zanette G (2021). Pregnancy and dentistry: a literature review on risk management during dental surgical procedures. Dentistry Journal.

[ref-49] FDA (1979). Prescription drug advertising: content and format for labeling of human prescription drugs. Federal Register.

[ref-50] FDA (2021). 2% LIDOCAINE HCI-2% lidocaine hci injection, solution. https://www.accessdata.fda.gov/spl/data/bd960807-498a-3434-e053-2a95a90acdaa/bd960807-498a-3434-e053-2a95a90acdaa.xml.

[ref-51] FDA (2022). Adrenalin® (Epinephrine injection, USP) 1mg/mL 1:1000 vial highlights of prescribing information. https://www.accessdata.fda.gov/spl/data/a821cf82-b4da-0ee1-e053-2a95a90a2ab2/a821cf82-b4da-0ee1-e053-2a95a90a2ab2.xml.

[ref-52] FDA (2023). ARTICAINE-articaine hydrochloride and epinephrine injection, solution. https://www.accessdata.fda.gov/spl/data/13158c3b-86a2-441a-b4d6-aaae2382d0ae/13158c3b-86a2-441a-b4d6-aaae2382d0ae.xml#S8.1.

[ref-53] Figuero E, Han YW, Furuichi Y (2020). Periodontal diseases and adverse pregnancy outcomes: mechanisms. Periodontology 2000.

[ref-54] Fisher SC, Siag K, Howley MM, Van Zutphen AR, Reefhuis J, Browne ML (2020). Maternal surgery and anesthesia during pregnancy and risk of birth defects in the National Birth Defects Prevention Study, 1997–2011. Birth Defects Research.

[ref-55] Fleisher Lee A, Fleischmann Kirsten E, Auerbach Andrew D, Barnason Susan A, Beckman Joshua A, Bozkurt B, Davila-Roman Victor G, Gerhard-Herman Marie D, Holly Thomas A, Kane Garvan C, Marine Joseph E, Nelson MT, Spencer Crystal C, Thompson A, Ting Henry H, Uretsky Barry F, Wijeysundera Duminda N (2014). 2014 ACC/AHA guideline on perioperative cardiovascular evaluation and management of patients undergoing noncardiac surgery. Journal of the American College of Cardiology.

[ref-56] Fomete B, Agbara R, Omeje KU, Oguntayo AO (2021). Odontogenic cervicofacial infection in pregnancy: a need for oral care. Journal of Family & Reproductive Health.

[ref-57] Franz-Montan M, Baroni D, Brunetto G, Sobral VR, da Silva CM, Venâncio P, Zago PW, Cereda CM, Volpato MC, de Araújo DR, de Paula E, Groppo FC (2015). Liposomal lidocaine gel for topical use at the oral mucosa: characterization, in vitro assays and in vivo anesthetic efficacy in humans. Journal of Liposome Research.

[ref-58] Fujita K, Sunada K (2021). Effect of epinephrine on the distribution of ropivacaine and lidocaine using radioactive isotopes in rat maxilla and pulp. Odontology.

[ref-59] Garret-Bernardin A, Cantile T, D’Antò V, Galanakis A, Fauxpoint G, Ferrazzano GF, De Rosa S, Vallogini G, Romeo U, Galeotti A (2017). Pain experience and behavior management in pediatric dentistry: a comparison between traditional local anesthesia and the wand computerized delivery system. Pain Research and Management.

[ref-60] Gazal G, Bahabri R, Alolayan AB, Alkayyal M, Al-Ghamdi R, Salamah R (2020). How successful is supplemental intraseptal and buccal infiltration anaesthesia in the mandibular molars of patients undergoing root canal treatment or tooth extraction?. Journal of Oral and Maxillofacial Research.

[ref-61] George A, Dahlen HG, Blinkhorn A, Ajwani S, Bhole S, Ellis S, Yeo A, Elcombe E, Johnson M (2018). Evaluation of a midwifery initiated oral health-dental service program to improve oral health and birth outcomes for pregnant women: a multi-centre randomised controlled trial. International Journal of Nursing Studies.

[ref-62] Giovannitti JA, Rosenberg MB, Phero JC (2013). Pharmacology of local anesthetics used in oral surgery. Oral and Maxillofacial Surgery Clinics of North America.

[ref-63] González-Jaranay M, Téllez L, Roa-López A, Gómez-Moreno G, Moreu G (2017). Periodontal status during pregnancy and postpartum. PLOS ONE.

[ref-64] Guastella C, Rinaldi V, Di Pasquale D, Coviello D, Pignataro L (2017). Oral pyogenic granuloma gravidarum: a case report describing a large bleeding lingual lesion. Journal of Obstetrics and Gynaecology: The Journal of the Institute of Obstetrics and Gynaecology.

[ref-65] Guimaraes CC, Lopes LC, Bergamaschi CC, Ramacciato JC, Silva MT, Araújo JO, de Andrade NK, Motta RHL (2021). Local anaesthetics combined with vasoconstrictors in patients with cardiovascular disease undergoing dental procedures: systematic review and meta-analysis. BMJ Open.

[ref-66] Guimicheva B, Czuprynska J, Arya R (2015). The prevention of pregnancy-related venous thromboembolism. British Journal of Haematology.

[ref-67] Guo J, Yin K, Roges R, Enciso R (2018). Efficacy of sodium bicarbonate buffered versus non-buffered lidocaine with epinephrine in inferior alveolar nerve block: a meta-analysis. Journal of Dental Anesthesia and Pain Medicine.

[ref-68] Gupta A, Sahai A, Aggarwal V, Mehta N, Abraham D, Jala S, Singh A (2021). Anesthetic efficacy of primary and supplemental buccal/lingual infiltration in patients with irreversible pulpitis in human mandibular molars: a systematic review and meta-analysis. Journal of Dental Anesthesia and Pain Medicine.

[ref-69] Gupta A, Wadhwa J, Aggarwal V, Mehta N, Abraham D, Aneja K, Singh A (2022). Anesthetic efficacy of supplemental intraligamentary injection in human mandibular teeth with irreversible pulpitis: a systematic review and meta-analysis. Journal of Dental Anesthesia and Pain Medicine.

[ref-70] Habak PJ, Griggs JRP (2022). Urinary tract infection in pregnancy. StatPearls.

[ref-71] Hagai A, Diav-Citrin O, Shechtman S, Ornoy A (2015). Pregnancy outcome after in utero exposure to local anesthetics as part of dental treatment: a prospective comparative cohort study. Journal of the American Dental Association.

[ref-72] Hameed NN, Sargod SS, Bhat SS, Hegde SK, Bava MM (2018). Effectiveness of precooling the injection site using tetrafluorethane on pain perception in children. Journal of Indian Society of Pedodontics and Preventive Dentistry.

[ref-73] Health MDoP (2016). Massachusetts oral health practice guidelines for pregnancy and early childhood. https://files.hria.org/files/PP2837.pdf.

[ref-74] Hindocha N, Manhem F, Bäckryd E, Bågesund M (2019). Ice versus lidocaine 5% gel for topical anaesthesia of oral mucosa—a randomized cross-over study. BMC Anesthesiology.

[ref-75] Hood D, Dewan D, James F (1986). Maternal and fetal effects of epinephrine in gravid ewes. Anesthesiology.

[ref-76] Hu W, Wang Y, Chen R, Pan T (2022). Oral health status and literacy/knowledge amongst pregnant women in Shanghai. International Dental Journal.

[ref-77] Iheozor-Ejiofor Z, Middleton P, Esposito M, Glenny A (2017). Treating periodontal disease for preventing adverse birth outcomes in pregnant women. Cochrane Database of Systematic Reviews.

[ref-78] Ilyas AM, Jennings JD, Banner L, Matzon JL (2020). Wide-awake surgery with local anesthesia and epinephrine is safe. Orthopedics.

[ref-79] Ince I, Arı MA, Dostbil A, Yalcin EK, Ozmen O, Khan MZ, Shimada T, Aksoy M, Tuncer K (2021). Does local anesthetic temperature affect the onset and duration of ultrasound-guided infraclavicular brachial plexus nerve block?: a randomized clinical trial. Brazilian Journal of Anesthesiology (English Edition).

[ref-80] Iqbal A, Lakkappa L, Chhabra P, Kondreddy K, Kumari S, Raju BM, Francis M (2022). Impact of chronic periodontitis on intrauterine growth of the fetus: an original research. Journal of Pharmacy and Bioallied Sciences.

[ref-81] Jamil FA, Asmael HM, Al-Jarsha MY (2020). The success of using 2% lidocaine in pain removal during extraction of mandibular premolars: a prospective clinical study. BMC Oral Health.

[ref-82] Jarvis SS, Shibata S, Bivens TB, Okada Y, Casey BM, Levine BD, Fu Q (2012). Sympathetic activation during early pregnancy in humans. The Journal of Physiology.

[ref-83] Jayasuriya NSS, Weerapperuma ID, Amarasinghe M (2017). The use of an iced cotton bud as an effective pre-cooling method for palatal anaesthesia: a technical note. Singapore Dental Journal.

[ref-84] Jimson S, Ranjani SS, Lenka S, Jimson S (2015). Comparative effects of clonidine and adrenaline with lignocaine during maxillary infiltration anaesthesia for dental extraction. Journal of Clinical and Diagnostic Research.

[ref-85] Johnson AP, Boscoe E, Cabrera-Muffly C (2020). Local blocks and regional anesthesia in the head and neck. Otolaryngologic Clinics of North America.

[ref-86] Joshi S, Bhate K, Kshirsagar K, Pawar V, Kakodkar P (2021). DentalVibe reduces pain during the administration of local anesthetic injection in comparison to 2% lignocaine gel: results from a clinical study. Journal of Dental Anesthesia and Pain Medicine.

[ref-87] Kapila Y (2021). Oral health’s inextricable connection to systemic health: special populations bring to bear multimodal relationships and factors connecting periodontal disease to systemic diseases and conditions. Periodontology 2000.

[ref-88] Karm MH, Kim M, Park FD, Seo KS, Kim HJ (2018). Comparative evaluation of the efficacy, safety, and hemostatic effect of 2% lidocaine with various concentrations of epinephrine. Journal of Dental Anesthesia and Pain Medicine.

[ref-89] Karm M, Park F, Kang M, Kim H, Kang J, Kim S, Kim Y, Kim C, Seo K, Kwon K, Kim C, Lee J, Hong S, Lim M, Nam S, Cho J (2017). Comparison of the efficacy and safety of 2% lidocaine HCl with different epinephrine concentration for local anesthesia in participants undergoing surgical extraction of impacted mandibular third molars: a multicenter, randomized, double-blind, crossover, phase IV trial. Medicine.

[ref-90] Kattan S, Lee SM, Hersh EV, Karabucak B (2019). Do buffered local anesthetics provide more successful anesthesia than nonbuffered solutions in patients with pulpally involved teeth requiring dental therapy?: a systematic review. Journal of the American Dental Association.

[ref-91] Kc K, Bhattarai BP, Subedi S (2022). Comparison of anesthetic efficacy of intraosseous injection with conventional inferior alveolar nerve block in mandibular third molar surgery: a systematic review and meta-analysis. Oral Surgery, Oral Medicine, Oral Pathology and Oral Radiology.

[ref-92] Kohlhepp LM, Hollerich G, Vo L, Hofmann-Kiefer K, Rehm M, Louwen F, Zacharowski K, Weber CF (2018). Physiological changes during pregnancy. Der Anaesthesist.

[ref-93] Kolding L, Eken H, Uldbjerg N (2020). Drug exposure during pregnancy and fetal cardiac function—a systematic review. Journal of Perinatal Medicine.

[ref-94] Kurien RS, Goswami M, Singh S (2018). Comparative evaluation of anesthetic efficacy of warm, buffered and conventional 2% lignocaine for the success of inferior alveolar nerve block (IANB) in mandibular primary molars: a randomized controlled clinical trial. Journal of Dental Research, Dental Clinics, Dental Prospects.

[ref-95] Lakshmanan L, Ravindran V (2021). Efficacy of cryotherapy application on the pain perception during intraoral injection: a randomized controlled trial. International Journal of Clinical Pediatric Dentistry.

[ref-96] Lato K, Bekes I, Widschwendter P, Friedl TWP, Janni W, Reister F, Froeba G, Friebe-Hoffmann U (2018). Hypotension due to spinal anesthesia influences fetal circulation in primary caesarean sections. Archives of Gynecology and Obstetrics.

[ref-97] Lee S, Chien D, Huang C, Shih S, Lee W, Chang W (2017). Dyspnea in pregnancy. Taiwanese Journal of Obstetrics & Gynecology.

[ref-98] Lee R, Milgrom P, Huebner C, Conrad D (2010). Dentists’ perceptions of barriers to providing dental care to pregnant women. Women’s Health Issues: Official Publication of the Jacobs Institute of Women’s Health.

[ref-99] Lee J, Shin T (2017). Use of local anesthetics for dental treatment during pregnancy; safety for parturient. Journal of Dental Anesthesia and Pain Medicine.

[ref-100] Lim ET, Chong KY, Singh B, Jong W (1992). Use of warm local anaesthetic solution for caudal blocks. Anaesthesia and Intensive Care.

[ref-101] Lim E, Mouyis M, MacKillop L (2021). Liver diseases in pregnancy. Clinical Medicine.

[ref-102] Liu PP, Wen W, Yu KF, Gao X, Wong MCM (2019). Dental care-seeking and information acquisition during pregnancy: a qualitative study. International Journal of Environmental Research and Public Health.

[ref-103] MacDonald E, Drum M, Nusstein J, Fowler S, Beck M, Reader A (2021). Anesthetic success using nitrous oxide and a combination of lidocaine/clonidine for the inferior alveolar nerve block and the effects on blood pressure and pulse in patients with symptomatic irreversible pulpitis: a prospective, randomized, double-blind study. Journal of Endodontics.

[ref-104] Mahapatra A, Nayak R, Satpathy A, Pati B, Mohanty R, Mohanty G, Beura R (2021). Maternal periodontal status, oral inflammatory load, and systemic inflammation are associated with low infant birth weight. Journal of Periodontology.

[ref-105] Maia FPA, Araujo Lemos CA, de Souza Andrade ES, de Morais SLD, do Egito Vasconcelos BC, Pellizzer EP (2022). Does the use of topical anesthetics reduce the perception of pain during needle puncture and anesthetic infiltration? Systematic review and meta-analysis of randomized controlled trials. International Journal of Oral and Maxillofacial Surgery.

[ref-106] Malamed SF (2014). Handbook of local anesthesia-e-book.

[ref-107] Manautou MA, Mayberry ME (2023). Local anesthetics and pregnancy. A review of the evidence and why dentists should feel safe to treat pregnant people. Journal of Evidence-Based Dental Practice.

[ref-108] Mark AM (2021). Pregnancy and oral health. Journal of the American Dental Association.

[ref-109] Massoth C, Chappell D, Kranke P, Wenk M (2022). Supine hypotensive syndrome of pregnancy: a review of current knowledge. European Journal of Anaesthesiology.

[ref-110] Mata K, Nobre A, Felix Silva P, Oliezer R, Fernandes C, Amaral J, Ramos J, Constante Gabriel Del-Arco M, Messora M, Tanus-Santos J, Gerlach R, Salvador S (2021). A new mixed model of periodontitis-induced preeclampsia: a pilot study. Journal of Periodontal Research.

[ref-111] Michalowicz BS, Diangelis AJ, Novak MJ, Buchanan W, Papapanou PN, Mitchell DA, Curran AE, Lupo VR, Ferguson JE, Bofill J (2008). Examining the safety of dental treatment in pregnant women. Journal of the American Dental Association.

[ref-112] Midha V, Midha V, Dua R, Garewal R, Kochhar AS, Kochhar GK (2021). Auxiliary aids to alleviate pain and anxiety during local anesthesia administration: a comparative study. International Journal of Clinical Pediatric Dentistry.

[ref-113] Moore PA (2016). An increased rate for major birth anomalies was not found following dental treatment requiring local anesthesia during pregnancy. Journal of Evidence Based Dental Practice.

[ref-114] Nafisi S, Samadi N, Houshiar M, Maibach HI (2018). Mesoporous silica nanoparticles for enhanced lidocaine skin delivery. International Journal of Pharmaceutics.

[ref-115] Nanitsos E, Vartuli R, Forte A, Dennison PJ, Peck CC (2009). The effect of vibration on pain during local anaesthesia injections. Australian Dental Journal.

[ref-116] Ogawa H, Kusumoto J, Nomura T, Hashikawa K, Terashi H, Sakakibara S (2021). Wire myography for continuous estimation of the optimal concentration of topical lidocaine as a vasodilator in microsurgery. Journal of Reconstructive Microsurgery.

[ref-117] OHRC (2023). Oral health care during pregnancy: a national consensus statement. https://www.mchoralhealth.org/materials/consensus_statement.php.

[ref-118] Ou YH, Ou YH, Gu J, Kang L (2019). Personalized anesthetic patches for dental applications. International Journal of Bioprinting.

[ref-119] Ouanounou A, Haas D (2016). Drug therapy during pregnancy: implications for dental practice. British Dental Journal.

[ref-120] Ouchi K, Jinnouchi A (2021). Calcium channel blockers, angiotensin II receptor antagonists and alpha-blockers accentuate blood pressure reducing caused by dental local anesthesia. Clinical Oral Investigations.

[ref-121] Papademetriou V, Stavropoulos K, Patoulias D, Papadopoulos C, Georgios K, Toumpourleka M, Sachinidis A (2021). Hypertension in pregnancy: unanswered questions. Current Pharmaceutical Design.

[ref-122] Patel D, Lahiri B, El-Patal MA-E, Alazmah A, Patel P, Abokhlifa YH (2021). To compare and analyze the potency of two topical anesthetic gels in reducing inferior alveolar injection pain in children of 8–12 years: a double-blinded clinical trial. Journal of Pharmacy and Bioallied Sciences.

[ref-123] Patil PM, Patil SP (2012). Is clonidine an adequate alternative to epinephrine as a vasoconstrictor in patients with hypertension?. Journal of Oral and Maxillofacial Surgery.

[ref-124] Pemathilaka RL, Reynolds DE, Hashemi NN (2019). Drug transport across the human placenta: review of placenta-on-a-chip and previous approaches. Interface Focus.

[ref-125] Phipps E, Prasanna D, Brima W, Jim B (2016). Preeclampsia: updates in pathogenesis, definitions, and guidelines. Clinical Journal of the American Society of Nephrology.

[ref-126] Phyo HE, Chaiyasamut T, Kiattavorncharoen S, Pairuchvej V, Bhattarai BP, Wongsirichat N (2020). Single buccal infiltration of high concentration lignocaine versus articaine in maxillary third molar surgery. Journal of Dental Anesthesia and Pain Medicine.

[ref-127] Purisch SE, Gyamfi-Bannerman C (2017). Epidemiology of preterm birth. Seminars in Perinatology.

[ref-128] Qi Y, Yao X, Du X, An S (2021). Local anesthetic lidocaine-encapsulated polymyxin-chitosan nanoparticles delivery for wound healing: in vitro and in vivo tissue regeneration. Drug Delivery.

[ref-129] Raju K, Berens L (2021). Periodontology and pregnancy: an overview of biomedical and epidemiological evidence. Periodontology 2000.

[ref-130] Ralston D, Shnider S (1978). The fetal and neonatal effects of regional anesthesia in obstetrics. Anesthesiology.

[ref-131] Rana S, Lemoine E, Granger JP, Karumanchi SA (2019). Preeclampsia: pathophysiology, challenges, and perspectives. Circulation Research.

[ref-132] Rayati F, Haeri M, Norouziha A, Jabbarian R (2021). Comparison of the efficacy of 4% articaine with epinephrine 1:100,000 and 2% lidocaine with epinephrine 1:100,000 buccal infiltration for single maxillary molar extraction: a double-blind, randomised, clinical trial. British Journal of Oral and Maxillofacial Surgery.

[ref-133] Ribeiro LN, Franz-Montan M, Breitkreitz MC, Alcântara AC, Castro SR, Guilherme VA, Barbosa RM, de Paula E (2016). Nanostructured lipid carriers as robust systems for topical lidocaine-prilocaine release in dentistry. European Journal of Pharmaceutical Sciences.

[ref-134] Ribeiro LNM, Franz-Montan M, Breitkreitz MC, Rodrigues da Silva GH, de Castro SR, Guilherme VA, de Araújo DR, de Paula E (2018). Nanohybrid hydrogels designed for transbuccal anesthesia. International Journal of Nanomedicine.

[ref-135] Ritchie HE, Oakes DJ, Kennedy D, Polson JW (2017). Early gestational hypoxia and adverse developmental outcomes. Birth Defects Research.

[ref-136] Rocha JS, Arima LY, Werneck RI, Moysés SJ, Baldani MH (2018). Determinants of dental care attendance during pregnancy: a systematic review. Caries Research.

[ref-137] Roh J, Han M, Kim KN, Kim KM (2016). The in vitro and in vivo effects of a fast-dissolving mucoadhesive bi-layered strip as topical anesthetics. Dental Materials Journal.

[ref-138] Salma RG, Alsayeh A, Maneea AB, Alrassan F, Almarshad A (2021). The effectiveness of electronic pulsed soft tissue vibration compared with topical anaesthesia in reducing the pain of injection of local anaesthetics in adults: a randomized controlled split-mouth clinical trial. International Journal of Oral and Maxillofacial Surgery.

[ref-139] Sandilya V, Andrade NN, Mathai PC, Aggarwal N, Sahu V, Nerurkar S (2019). A randomized control trial comparing buccal infiltration of 4% articaine with buccal and palatal infiltration of 2% lignocaine for the extraction of maxillary premolar teeth. Contemporary Clinical Dentistry.

[ref-140] Sarwal P, Lapumnuaypol K (2022). Pyogenic granuloma. StatPearls.

[ref-141] Sato Y, Ikoma T, Wakita R, Fukayama H (2019). Interfacial interaction of anesthetic lidocaine and mesoporous silica nanoparticles in aqueous solutions and its release properties. Journal of Materials Chemistry B.

[ref-142] Schwartz N, Nachum Z, Green M (2015). The prevalence of gestational diabetes mellitus recurrence--effect of ethnicity and parity: a metaanalysis. American Journal of Obstetrics and Gynecology.

[ref-143] Senthoor P, Janani K, Ravindran C (2020). A prospective, randomized double-blinded study to evaluate the efficacy of buffered local anesthetics in infected and inflamed pulp and periapical tissues. Journal of Maxillofacial and Oral Surgery.

[ref-144] Shadmehr E, Aminozarbian MG, Akhavan A, Mahdavian P, Davoudi A (2017). Anaesthetic efficacy of lidocaine/clonidine for inferior alveolar nerve block in patients with irreversible pulpitis. International Endodontic Journal.

[ref-145] Shadmehr E, Sarmast ND, Davoudi A, Chung YJ, Wang HH (2021). The additive effect of clonidine to lidocaine on postoperative pain management after root canal treatment on mandibular molars with symptomatic irreversible pulpitis: a prospective randomised double-blind clinical trial. Journal of Conservative Dentistry.

[ref-146] Silva de Araujo Figueiredo C, Gonçalves Carvalho Rosalem C, Costa Cantanhede AL, breu Fonseca Thomaz ÉB, Fontoura Nogueira da Cruz MC (2017). Systemic alterations and their oral manifestations in pregnant women. Journal of Obstetrics and Gynaecology Research.

[ref-147] Singla M, Gugnani M, Grewal MS, Kumar U, Aggarwal V (2022). Does the presence and amount of epinephrine in 2% lidocaine affect its anesthetic efficacy in the management of symptomatic maxillary molars with irreversible pulpitis?. Journal of Dental Anesthesia and Pain Medicine.

[ref-148] Smereka J, Aluchna M, Aluchna A, Puchalski M, Wroblewski P, Checinski I, Leskiewicz M, Szarpak L (2019). Medical emergencies in dental hygienists’ practice. Medicine.

[ref-149] Sokhov ST, Rabinovich SA, Bogaevskaya OY (2019). Comparative evaluation of P.M. Egorov and modified G. Gow-Gates mandibular block efficacy. Stomatologiia.

[ref-150] Southerland JH, Gill DG, Gangula PR, Halpern LR, Cardona CY, Mouton CP (2016). Dental management in patients with hypertension: challenges and solutions. Clinical, Cosmetic and Investigational Dentistry.

[ref-151] Sovatdy S, Vorakulpipat C, Kiattavorncharoen S, Saengsirinavin C, Wongsirichat N (2018). Inferior alveolar nerve block by intraosseous injection with Quicksleeper® at the retromolar area in mandibular third molar surgery. Journal of Dental Anesthesia and Pain Medicine.

[ref-152] Spivakovsky S (2019). Injectable local anaesthetic agents for dental anaesthesia. Evidence-Based Dentistry.

[ref-153] St George G, Morgan A, Meechan J, Moles DR, Needleman I, Ng YL, Petrie A (2018). Injectable local anaesthetic agents for dental anaesthesia. Cochrane Database of Systematic Reviews.

[ref-154] Takahashi Y, Nakano M, Sano K, Kanri T (2005). The effects of epinephrine in local anesthetics on plasma catecholamine and hemodynamic responses. Odontology.

[ref-155] Tamirisa KP, Elkayam U, Briller JE, Mason PK, Pillarisetti J, Merchant FM, Patel H, Lakkireddy DR, Russo AM, Volgman AS, Vaseghi M (2022). Arrhythmias in pregnancy. JACC: Clinical Electrophysiology.

[ref-156] Tan E, Tan E (2013). Alterations in physiology and anatomy during pregnancy. Best Practice & Research Clinical Obstetrics & Gynaecology.

[ref-157] Tanaka E, Yoshida K, Kawaai H, Yamazaki S (2016). Lidocaine concentration in oral tissue by the addition of epinephrine. Anesthesia Progress.

[ref-158] Tangen LF, Lundbom JS, Skarsvåg TI, Wågø KJ, Ballo S, Hjelseng T, Finsen V (2016). The influence of injection speed on pain during injection of local anaesthetic. Journal of Plastic Surgery and Hand Surgery.

[ref-159] Taylor NAS, Nykvist Å, Powers N, Caldwell JN (2019). Thermoeffector threshold plasticity: the impact of thermal pre-conditioning on sudomotor, cutaneous vasomotor and thermogenic thresholds. Journal of Thermal Biology.

[ref-160] Tettamanti L (2017). Pregnancy and periodontal disease: does exist a two-way relationship?. Oral & Implantology.

[ref-161] Tirupathi SP, Rajasekhar S (2020). Effect of warming local anesthesia solutions before intraoral administration in dentistry: a systematic review. Journal of Dental Anesthesia and Pain Medicine.

[ref-162] Tolcher MC, Fisher WE, Clark SL (2018). Nonobstetric surgery during pregnancy. Obstetrics & Gynecology.

[ref-163] Tomlin PJ (1974). Death in outpatient dental anaesthetic practice. Anaesthesia.

[ref-164] Tschopp C, Tramèr M, Schneider A, Zaarour M, Elia N (2018). Benefit and harm of adding epinephrine to a local anesthetic for neuraxial and locoregional anesthesia: a meta-analysis of randomized controlled trials with trial sequential analyses. Anesthesia & Analgesia.

[ref-166] Uppal NN, Jhaveri M, Hong S, Shore-Lesserson L, Jhaveri KD, Izzedine H (2022). Local anesthetics for the Nephrologist. Clinical Kidney Journal.

[ref-167] van Hove H, Mathiesen L, Freriksen J, Vähäkangas K, Colbers A, Brownbill P, Greupink R (2022). Placental transfer and vascular effects of pharmaceutical drugs in the human placenta ex vivo: a review. Placenta.

[ref-168] Vasco Ramirez M, Valencia GC (2020). Anesthesia for nonobstetric surgery in pregnancy. Clinical Obstetrics & Gynecology.

[ref-169] Vigarios E, Maret D (2020). Pregnancy gingivitis. QJM: an International Journal of Medicine.

[ref-170] Visconti RP, Tortamano IP, Buscariolo IA (2016). Comparison of the anesthetic efficacy of mepivacaine and lidocaine in patients with irreversible pulpitis: a double-blind randomized clinical trial. Journal of Endodontics.

[ref-171] Wadia R (2022). Supplemental intraligamentary injections. British Dental Journal.

[ref-172] Wali M, Drum M, Reader A, Nusstein J (2010). Prospective, randomized single-blind study of the anesthetic efficacy of 1.8 and 3.6 milliliters of 2% lidocaine with 1:50,000 epinephrine for inferior alveolar nerve block. Journal of Endodontics.

[ref-173] Wang YH, Wang DR, Liu JY, Pan J (2021). Local anesthesia in oral and maxillofacial surgery: a review of current opinion. Journal of Dental Sciences.

[ref-174] Whelehan A, Delanty N (2019). Therapeutic strategies for treating epilepsy during pregnancy. Expert Opinion on Pharmacotherapy.

[ref-175] WHO (2017). WHO recommendations on maternal health. https://apps.who.int/iris/bitstream/handle/10665/259268/WHO-MCA-17.10-eng.pdf;jsessionid=57C08E4FA77A7D864CBE8E59500D3590?sequence=1.

[ref-176] Yadav S (2015). Anesthetic success of supplemental infiltration in mandibular molars with irreversible pulpitis: a systematic review. Journal of Conservative Dentistry.

[ref-177] Yanamandra N, Chandraharan E, Chandraharan E, Arulkumaran S (2012). Anatomical and physiological changes in pregnancyand their implications in clinical practice. Obstetric and Intrapartum Emergencies: a Practical Guide to Management.

[ref-178] Yilmaz K, Tunga U, Ozyurek T (2018). Buccal infiltration versus inferior alveolar nerve block in mandibular 2(nd) premolars with irreversible pulpitis. Nigerian Journal of Clinical Practice.

[ref-179] Yousefi M, Parvaie P, Riahi S (2020). Salivary factors related to caries in pregnancy: a systematic review and meta-analysis. Journal of the American Dental Association.

[ref-180] Zanjir M, Lighvan NL, Yarascavitch C, Beyene J, Shah PS, Azarpazhooh A (2019). Efficacy and safety of pulpal anesthesia strategies during endodontic treatment of permanent mandibular molars with symptomatic irreversible pulpitis: a systematic review and network meta-analysis. Journal of Endodontics.

[ref-181] Zhao H, Wong RJ, Stevenson DK (2021). The impact of hypoxia in early pregnancy on placental cells. International Journal of Molecular Sciences.

[ref-182] Zhou C, Tang L, Yin Q, Yang L, Gong D, Kang Y, Cao H, Fan J, Zhang Y, Qian D, Zhang Q, Ke B, Liu J, Zhang W, Yang J (2020). Novel compound LL-a produces long and nociceptive-selective regional anesthesia via TRPV1 channels in rodents sciatic nerve block model. Regional Anesthesia & Pain Medicine.

